# Notes and Notices

**Published:** 1899-05

**Authors:** 


### NOTES AND NOTICES.

See a woman, in another column, picking grapes for Speer's wines. Read all about it. Excellent for weakly persons and the aged, particularly for females.

Dr. J. Henry Allen will remove his residence on May 2d, to No. 219 East Forty-second Place, near Cottage Grove Avenue, Chicago, Illinois. Telephone, Oakland 492.

Endorsement of Speer's wines by the Faculty of N. Y.—Dr. Cyrus Edson of New York Board of Health, says there are no better wines in the world.

Dr. John Storer wishes to announce his removal on May 1st, from 809 Columbus Memorial Building to 1007 Columbus Memorial Building, Chicago, Illinois. Office hours, 10 A. M. to 1 P. M. Telephone, Main 4575. Residence, 108 South Oak Park Avenue, Oak Park, Ill. Hours: 8 to 9 A. M.; 5 to 7 P. M. Sundays: 12 to 1. Telephone 1272.

N. Y. Board of Health on Wine.—Dr. Janes, of the New York Board of Health, says: "I take pleasure in testifying to the superior qualities of the wine produced by Alfred Speer. I recommend it as a superior wine for the sick and debilitated." The Port and Burgundy have no equal.

The Homœopathic Medical Examining Board of Pennsylvania, June 20th, 21st, 22d, and 23d, 1899. Place of meeting, Philadelphia, Church of New Jerusalem, Twenty-second and Chestnut Streets. Members of the Board—Dr. Augustus Korndœrfer, president, Philadelphia; Dr. Joseph C. Guernsey, Secretary, 1923 Chestnut Street, Philadelphia; Dr. Isaac G. Smedley, Philadelphia; Dr. John F. Cooper, Pittsburg; Dr. John J. Detwiller, Easton; Dr. Edward Cranch, Erie; Dr. Lewis H. Willard, Allegheny.

See a woman, in another column, picking grapes for Speer's wines. Read all about it. Excellent for weakly persons and the aged, particularly for females.

### CASE OF CHRONIC PYROSIS.

Ella Sterrett, Long Island City, American, age thirty; first seen June 10th, 1898; suffering from chronic pyrosis, for which she had been under treatment for twenty-four years with only occasional temporary relief from this most painful condition. Almost everything generally known had been done for her, but without effective result. From the beginning of the complaint, in her sixth year, up to the age of twenty-eight, while it was a source of great pain and discomfort, it did not seem greatly to interfere with assimilation and nutrition. But at twenty-nine, it took on a more violent form: she became unable to retain anything on her stomach, unless preceded by cocaine combined with some other stomach sedative. Even this was but partially successful, as the stomach would often throw it all off in spite of the medicinal action. After her twenty-ninth year, she began to lose flesh rapidly; so that her weight had already been reduced within the last year from 160 to 83 pounds. When first seen she presented every appearance of malnutrition and debility. Her bowels would sometimes not move for a week, in spite of cathartics and hot water injections.

The patient was now put to bed and given the following treatment: first the stomach was washed out with a weak Thiersch solution, and ten drops of bovinine in iced grape juice were given every hour, extending through the night. After twenty-four hours, she said her stomach felt greatly relieved. The bovinine was then increased to a teaspoonful in a wineglassful of milk every two hours; the first two doses being thrown off, but the third and subsequent ones were retained, without discomfort. This was continued until the 10th, with steady improvement, when I deemed it wise to suggest an operation for a relief of the constipation, as the bowels refused to respond to any but the most drastic cathartics, and these being weakening and irritating, it seemed not wise to continue them. Patient finally consented to operation, and on the 16th, under chloroform, the rectum and anus were thoroughly stretched and the bowel washed high up with soap-suds and water. This was followed by a most copious evacuation. She reacted nicely from the anæsthetic, and since then the bowels moved regularly once a day. On the 18th, the bovinine was increased to a tablespoonful in milk every three hours. To this time, she had lived on nothing but the bovinine in milk, and through all she showed perfect nutrition and no loss of strength. After this she was allowed in addition some easily digested food. She increased in weight, and had no further attacks of the gastric colic. On the 29th, the bovinine was increased to a wineglassful in milk and grape juice alternately. July 7th, 1898, patient was discharged cured, at a weight of 124 pounds; a most remarkable and unparalleled gain of 41 pounds in less than a month.—*Modern Medical Science.*

